# Prevalence and aetiologies of anaemia among first trimester pregnant women in Sri Lanka; the need for revisiting the current control strategies

**DOI:** 10.1186/s12884-021-04341-z

**Published:** 2022-01-06

**Authors:** Gayani Shashikala Amarasinghe, Thilini Chanchala Agampodi, Vasana Mendis, Krishanthi Malawanage, Chamila Kappagoda, Suneth Buddhika Agampodi

**Affiliations:** 1 Department of Community Medicine, Faculty of Medicine and Allied Sciences, Rajarata University of Sri Lanka, Saliyapura, Sri Lanka; 2 Department of Pathology, Faculty of Medicine and Allied Sciences, Rajarata University of Sri Lanka, Saliyapura, Sri Lanka; 3 Regional Director of Health Services Office - Anuradhapura, Ministry of Health, Anuradhapura, Sri Lanka

**Keywords:** Anaemia, Pregnancy, Iron deficiency, Vitamin B12, Folate, South-east Asian Ovalocytosis, Sri Lanka

## Abstract

**Background:**

The Sustainable development goals, which focus strongly on equity, aim to end all forms of malnutrition by 2030. However, a significant cause of intergenerational transfer of malnutrition, anaemia in pregnancy, is still a challenge. It is especially so in the low- and middle-income settings where possible context-specific aetiologies leading to anaemia have been poorly explored. This study explores the prevalence of etiological factors significantly contributing to anaemia in pregnancy in Sri Lanka, a lower-middle-income country with a high prevalence of malnutrition albeit robust public health infrastructure.

**Methods:**

All first-trimester pregnant women registered in the public maternal care programme in the Anuradhapura district from July to September 2019 were invited to participate in Rajarata Pregnancy Cohort (RaPCo). After a full blood count analysis, high-performance liquid chromatography, peripheral blood film examination, serum B12 and folate levels were performed in anaemic participants, guided by an algorithm based on the red cell indices in the full blood count. In addition, serum ferritin was tested in a random subsample of 213 participants. Anaemic women in this subsample underwent B12 and folate testing.

**Results:**

Among 3127 participants, 14.4% (95%CI 13.2–15.7, *n* = 451) were anaemic. Haemoglobin ranged between 7.4 to 19.6 g/dl. 331(10.6%) had mild anaemia. Haemoglobin ≥13 g/dl was observed in 39(12.7%). Microcytic, normochromic-normocytic, hypochromic-normocytic and macrocytic anaemia was observed in 243(54%), 114(25.3%), 80(17.8%) and two (0.4%) of full blood counts in anaemic women, respectively. Microcytic anaemia with a red cell count ≥5 * 10^6^ /μl demonstrated a 100% positive predictive value for minor haemoglobinopathies. Minor hemoglobinopathies were present in at least 23.3%(*n* = 105) of anaemic pregnant women. Prevalence of iron deficiency, B12 deficiency and Southeast Asian ovalocytosis among the anaemic was 41.9% (95%CI 26.4–59.2), 23.8% (95%CI 10.6–45.1) and 0.9% (95%CI 0.3–2.3%), respectively. Folate deficiency was not observed.

**Conclusion:**

Even though iron deficiency remains the primary cause, minor hemoglobinopathies, B 12 deficiency and other aetiologies substantially contribute to anaemia in pregnancy in this study population. Public health interventions, including screening for minor hemoglobinopathies and multiple micronutrient supplementation in pregnancy, should be considered in the national programme for areas where these problems have been identified.

## Background

Anaemia in pregnancy is a major public health problem as many countries across the globe are struggling to quantify, prevent and treat this condition. The global prevalence of this issue is estimated to be around 38%, while 58.1% of South Asian pregnant women are thought to be anaemic [1]. The sustainable development goals call for ending all forms of malnutrition, including anaemia, by 2030, highlighting the need for mending the inequality gaps at global, national and subnational levels [2]. However, in low and middle-income countries (LMICs), slow and uneven progress in the prevention of anaemia is reported specifically due to complex context-specific aetiologies that are poorly identified [3].

Anaemia during pregnancy can adversely affect both the mother and the foetus. Severely anaemic pregnant women have a 2.36 times higher risk of death than other pregnant women [4] and are more vulnerable to adverse pregnancy outcomes [5]. Maternal anaemia also increases the baby’s risk for intellectual development problems, obesity, cardiac diseases and anaemia [6–9].

Traditionally the most typical cause of anaemia is iron deficiency, contributing to more than 50% of anaemia in pregnancy worldwide [10, 11]. The demand for iron is increased during pregnancy due to increased maternal erythropoiesis. Iron is also required for the growth of foetal tissues and iron stores [12–14]. When the supply fails to meet the increased demand due to various reasons such as poor diet, infections or infestations, iron stores in the body gradually depletes, manifesting as iron deficiency anaemia at some point. In addition to anaemia, iron deficiency can lead to maternal fatigue and hormonal derangements, and increase the risk of postpartum haemorrhage and depression [15–18]. It can also lead to iron deficiency in the offspring, especially when it is present during late pregnancy [19].

Other micronutrient deficiencies (like Folate, Vitamin B12, vitamin B6, vitamin A, vitamin C and Zinc), hemoglobinopathies, red cell membrane disorders, and systemic causes (such as hormonal disorders, liver diseases, malaria and Human Immunodeficiency Virus (HIV)) also contribute to anaemia in pregnancy [20, 21]. The contribution of each of these aetiologies to anaemia among pregnant women will vary in different communities. How different etiological factors would affect the mother’s overall health, and the baby is also diverse.

As an example, folate deficiency is high in malaria-endemic regions and in countries where the staple food is not fortified with folate [3]. Pregnant women are specially vulnerable to folate deficiency as its requirement is significantly increased in pregnancy [22]. Folate is essential for purine and thymidine nucleotide biosynthesis and homocysteine metabolism. Therefore, folate deficiency leads to DNA synthesis abnormalities, homocysteine elevation, and altered cellular gene expression [22]. Inhibited DNA synthesis affect mitosis causing ineffective erythropoiesis from hematopoietic precursor cells. Macrocytic anaemia, pancytopenia and hyper segmented neutrophils may become evident in the blood [23]. Reduction of red cell lifespan is another contributor to anaemia seen in folate deficiency [3]. The same mechanisms can lead to increased risk for congenital abnormalities (such as neural tube defects), foetal growth retardation, preterm delivery, and neonatal folate deficiency in addition to maternal anaemia [22].

Vitamin B12 deficiency is highly prevalent in some countries of the Indian subcontinent, where 32% of first trimester pregnant women are reportedly B12 deficient [24]. B12 deficiency has been reported among 15–49% of pregnant women in Latin America and the Caribbeans [25, 26]. Converting homocysteine and methyltetrahydrofolate to methionine and tetrahydrofolate requires Vitamin B12. In its deficiency, folate is trapped as methyltetrahydrofolate, causing a functional folate deficiency. Thus, pathophysiology of macrocytic anaemia and adverse foetal outcomes of B12 deficiency are similar to folate deficiency [27].

Anaemia due to hereditary conditions such as minor hemoglobinopathies or membrane disorders can be prevalent in some communities. For example, any variant of hemoglobinopathy is carried by 45.5% of the South Asian population [28]. Having a heterozygous variant haemoglobin phenotype increases the risk of anaemia in pregnancy and having a baby affected with the homozygous disease.

Successful control of anaemia in a pregnant woman requires alleviation of its aetiology. In many parts of the world, the focus of public health interventions to prevent and control anaemia in pregnancy is almost limited to controlling iron deficiency [29]. Multiple interventions across the lifecycle to build and maintain good iron stores have been adopted widely to prevent iron deficiency anaemia in pregnant women. Iron supplementation, treating anaemia with iron, deworming, and nutritional education are examples of such interventions [30].

Despite having a robust public health system catering for its population across the lifecycle, anaemia prevalence among pregnant women in Sri Lanka is 29.1% (according to routinely reported data of the reproductive health management information system (RHMIS)) [31], resembling one of the worst maternal health indicators in the country. However, many strategies have been adopted to prevent anaemia in pregnancy in the country. Early in the life cycle, iron and folate supplementation and clinical screening for anaemia are conducted at schools [32]. Universal anaemia screening with clinical and biochemical parameters is a standard component of care provided at pre conceptional clinics, first antenatal clinic visit and the clinic visit around 28 weeks of gestation. Universal folic acid supplementation is commenced pre conceptionally and continued throughout pregnancy until 6 months postpartum, coupled with iron supplementation after the first trimester of pregnancy. A food supplement, “Thriposha” (including macronutrients, iron, zinc, folic acid, vitamin B complex etc.), is provided for all pregnant and lactating women. Nutritional counselling, deworming, treating identified anaemia with double iron dose, and referral of refractory cases for further care are other interventions implemented under the country’s public health system [33, 34].

Amid the success of such strategies, other aetiologies gain prominence marking poor progress of anaemia control. Identification of aetiologies significantly contributing to anaemia in pregnancy in a particular community enables the implementation of policies and interventions to address them, leading to better control of anaemia and improved maternal and foetal outcomes. However, etiological assessment is often costly and less feasible in settings where the disease burden is highest, the LMICs. Categorising anaemia according to the red cell indices in the full blood count and using these categorisations to guide treatment and further assessment is helpful in the low resource settings.

This study aims to determine the actual prevalence of anaemia and its’ aetiologies among first trimester pregnant women in Anuradhapura district, Sri Lanka. To our knowledge, this is one of the largest studies on early maternal anaemia in LMICs and the first Sri Lankan study that assess the contribution of several important aetiologies to anaemia in pregnancy, which therefore would provide data for specific preventive strategies in vulnerable populations.

## Methods

This population-based study was carried out as a part of the Rajarata Pregnancy Cohort (RaPCo) in Anuradhapura district, Sri Lanka [35]. Anuradhapura district reported a 39.1% prevalence of anaemia in pregnancy, ranking fifth-highest among the 26 health districts of the country [31]. Anaemia prevalence at the time of the first antenatal visit was 22.8%, according to RHMIS data 2018.

Pregnant women newly registered in the national pregnancy care programme from July to September 2019 were invited for the study. We selected first trimester pregnant women (up to the completion of 13 weeks of gestation) for this component. The detailed methodology has been explained elsewhere [35, 36].

We hypothesised that causes of anaemia affecting at least 10% of anaemic pregnant women would be significant from a public health point of view. To detect an aetiology with a 10% prevalence (precision 0.03, 95% confidence level, 10% nonresponse rate), at least 425 anaemic pregnant women were needed to be included. Thus, according to the anaemia prevalence in the district, at least 1866 pregnant women were needed to be recruited.

Invitation to participate in the study was sent to all eligible participants through their Public Health Midwife. From July to October 2019, a special clinic was conducted fortnightly at each health division (Medical Officer of Health area), where the eligible participants were recruited consecutively. Altogether, 226 clinics were conducted across the district for participant recruitment.

An interviewer-administered questionnaire and a physical examination to elicit clinical signs were used for data collection. In participants reporting a previously diagnosed minor hemoglobinopathy, the HPLC report was observed for verification. Venepuncture was done to collect whole blood and serum samples and to prepare peripheral blood films. All slides were prepared in the field, and the serum separation was done within 2–4 h of sample collection. Aliquots of samples were stored at -80 °C.

We performed a full blood count for all the participants. Out of the calculated minimum sample, 10% was randomly selected for Serum ferritin testing to estimate iron status [37, 38]. Peripheral blood film analysis was also performed for the subsample. In addition to that, all anaemic women in this subsample were invited to undergo B12, Folate and HPLC testing.

According to current evidence, all the anaemic women (in the original sample) were classified into five mutually exclusive categories based on their red cell indices. When there was microcytic anaemia (MCV < 80 fl) with a high RBC index (RCC ≥ 5*10^6^_), minor hemoglobinopathy was suspected [39, 40]. They were referred to undergo High-Performance Liquid Chromatography (HPLC) [41]. When there was normocytic normochromic anaemia in a participant, a membrane disorder or mixed deficiency was suspected [42]. A peripheral blood film analysis was performed on them. B 12 or folate deficiency was suspected when there was macrocytic anaemia (MCV ≥96 fl) [43]. A peripheral blood film analysis was performed for them, and they were invited for Serum B12 and folate testing. When there was microcytic anaemia with a normal RCC, iron deficiency was suspected. When there was normocytic anaemia (MCV 80–95.9 fl) with hypochromic cells (MCH ≤27 pg), early iron deficiency was suspected [38].

### Definitions/measurements

Anaemia (within the first 13 weeks of gestation) was defined as having haemoglobin less than 11 g/dl [30]. Haemoglobin levels below 7 g/dl, between 7 to 9.9 g/dl and between 10 to 10.9 g/dl were used to classify severe, moderate and mild anaemia, respectively [44]. Ferritin levels equal to or below 30 ng/ml were considered as iron deficiency [38]. Serum Folate levels below 4 ng/ml and B12 less than 203 pg/ml were considered as deficiency in each nutrient [45].

### Laboratory investigations

Serum ferritin, Homocysteine, B12 and Folate assessments were performed in a commercial laboratory with external quality control methods. HPLC was performed at the thalassemia unit of Teaching hospital, Anuradhapura. Other investigations (complete blood count, peripheral blood film analysis and liver functions) were performed in a public health research laboratory with internal and external quality control. Samples for serum B12, folate and homocysteine were stored and transported refrigerated and covered from exposure to sunlight. They were analysed as soon as possible with a maximum stored time of 48 h.

### Data management and analysis

Data from the interviewer-administered questionnaire was entered directly into a cloud-based password secured database. Laboratory investigations data were later merged with the cloud data. The first author has manually verified a 20% of the full blood count data and all of the other laboratory investigation data used for this paper.

Data analysis was performed using SPSS version 22 and Open Epi. The graphs were prepared using GraphPad Prism and Microsoft word. Fissures exact test was performed to compare the proportions of self-reported thyroid, liver, renal and autoimmune diseases among anaemic and non-anaemic pregnant women.

## Results

Of the first trimester pregnant women registered in the public maternal care program in the district, 3127 (86% of the total population) were recruited for the anaemia study. The majority of the participants were ethnic Sinhalese (87%), and 31% were in their first pregnancy (Table 1). The mean age of the group was 28.3 years. A history of anaemia was reported by 595 (18.8%). Only 1857 (80.7%) participants reported that they were on folic acid supplementation before the pregnancy and 2977 (94.6%) were on folic acid at the time of the recruitment.

**Table 1:** Baseline characteristics of the 3127 first trimester pregnant women participating in the study

Baseline characteristic		n	%
Maternal age	< 20	205	6.5
	20–24	636	20.1
	25–29	1106	35.0
	30–34	782	24.7
	35–39	370	11.7
	<=40	65	2.1
Ethnicity	Sinhala	2720	87.0
	Moor/Malay	365	11.7
	Other	42	1.2
Religion	Buddhist	2715	86.0
	Islam	380	12.0
	Catholic/Christian	47	1.5
	Hindu	15	0.5
	Other	0	0.0
Education level	Up to G.C.E Ordinary level	1872	60.2
	G.C.E Ordinary to G.C.E Advanced level	477	15.3
	Certificates or diplomas	454	14.6
	Graduate	306	9.8
Gravidity	Primigravid	973	30.8
	Multigravida	2182	69.2
BMI	Underweight	509	16.6
	Normal	1002	32.7
	Overweight	490	16.0
	Obese	1064	34.7
History of anaemia	No	2519	79.6
	Yes, detected during an antenatal period of a previous pregnancy	380	12.0
	Yes, detected during postnatal period/ post-abortion of a previous pregnancy	56	1.8
	Yes, but not related to a pregnancy	159	5.0
Pre conceptional folic acid supplementation	Yes	1855	80.7
	No	443	19.3
Antenatal folic acid supplementation	Yes	2977	94.6
	No	169	5.4
History of diagnosed thyroid diseases	Yes	78	2.5
	No	3065	97.5
History of diagnosed liver diseases	Yes	7	0.2
	No	3136	99.8
History of diagnosed renal diseases	Yes	15	0.5
	No	3128	99.5
History of diagnosed autoimmune diseases	Yes	5	0.2
	No	3138	99.8

Haemoglobin was distributed between 7.4 to 19.6 g/dl, and the mean was 11.9 g/dl (SD 1.0). Mean MCV was 83.2 fl (SD 6.4). Mean RCC, MCH and MCHC were 4.51*10^6^ /μl (SD-0.41), 26.6 pg (SD-2.5) and 31.88(SD-0.93), respectively**.**

**Fig. 1:**
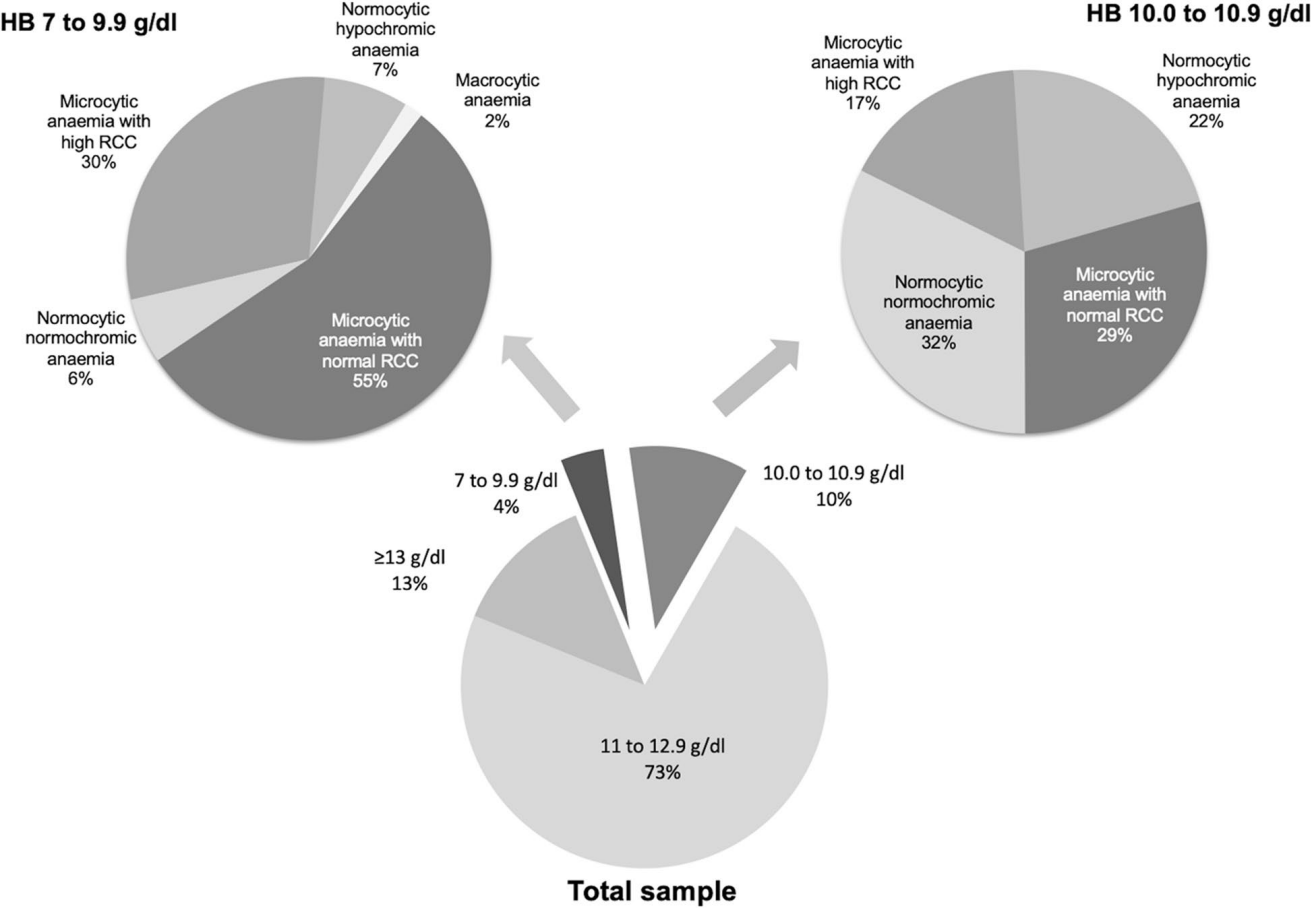
Distribution of haemoglobin levels and the types of anaemia among 3127 first trimester pregnant women

The prevalence of anaemia in the study sample was 14.4% (95% CI 13.2–15.7, *n* = 451). None of them had severe anaemia (Hb < 7 g/dl), and the majority (*n* = 331, 10.6%) had mild anaemia. A haemoglobin level of 13 g/dl or more was observed in 399 (12.7%). The types and severity of anaemia in the sample is shown in Fig. 1.

Microcytic red cells were seen among 638 (20.4%) of the participants. The majority of anaemic women (n-243, 54%) had microcytic anaemia. Of them, 91 had RCC more than 5 * 10^6^ /μl. This group accounted for 20.2% of the anaemic pregnant women, and their mean haemoglobin level was 10 g/dl (SE-0.1). The mean haemoglobin level in the group with microcytic anaemia and normal RCC was 9.9 g/dl (SE-0.1).

**Fig. 2:**
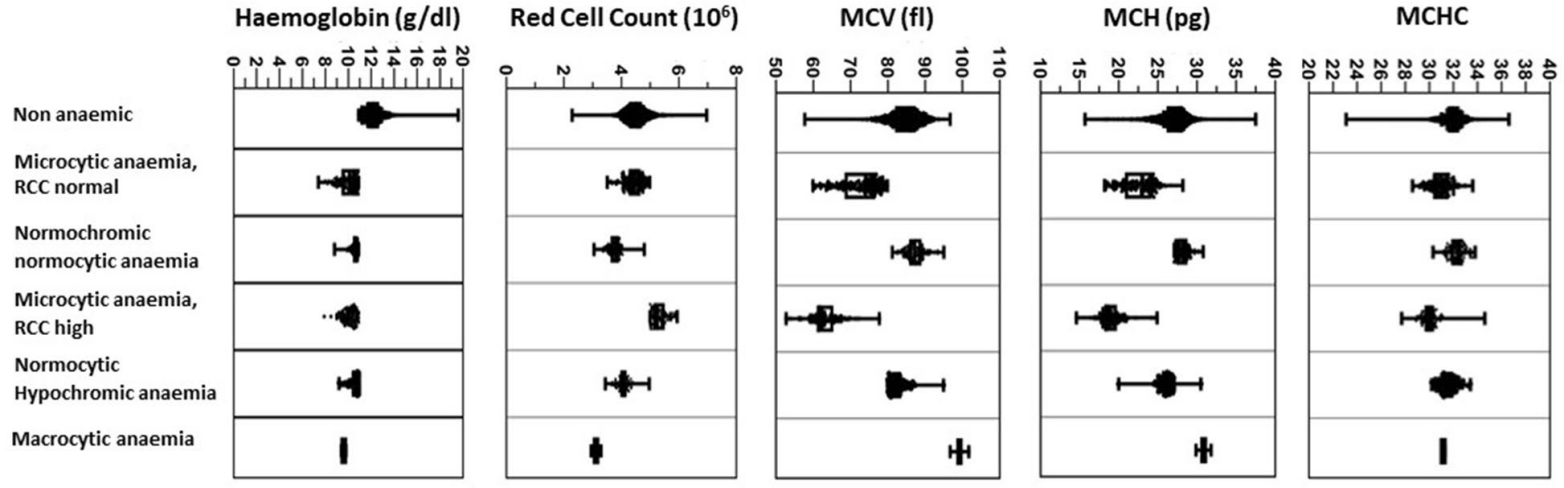
Distribution of Haemoglobin and red cell indices according to the type of anaemia among first trimester pregnant women

Normocytic normochromic anaemia was seen among 114 (25.3%) anaemic women. Their mean haemoglobin level was 10.6 g/dl. Normocytic hypochromic anaemia was seen in 80 (17.8%) anaemic women who had mean haemoglobin of 10.5 g/dl. Two (0.4%) of the anaemic women had macrocytic anaemia (mean haemoglobin level 9.6 g/dl). Distribution of Haemoglobin and Red cell indices; RCC, MCV, MCH, MCHC according to the type of anaemia is presented in Fig. 2.

### Microcytic anaemia with RCC < 5 × 10^6^ /μl

A small random subsample of 11 out of the 163 of them underwent peripheral blood film analysis. Minor hemoglobinopathy was seen in four, iron deficiency and early iron deficiency were seen in three each, and early megaloblastic changes were seen in one participant(s). HPLC reports were available in 21 (9 had records, 12 were tested during the current study), and 14 of them were confirmed as having thalassemia trait.

### Normocytic normochromic anaemia

A blood film reporting was available for 89 out of 114 women with normocytic normochromic anaemia (11 blood films were of poor quality and were excluded). Four cases (4.5%, CI 1.8–11.0%) of South-East Asian Ovalocytosis were detected. It contributed to 0.9% (95% CI 0.3 to 2.3%) of anaemia in first trimester women.

Features of iron deficiency, early mixed deficiency, mixed deficiency, early megaloblastic changes and megaloblastic changes were observed in 38 (40.9%), 18 (19.4%), 6 (6.5%), 7 (7.5%) and 1 (1.1%), respectively. 19 (20.4%) blood film reports were normal. Two pregnant women, the one with megaloblastic changes and another with early megaloblastic features in their blood films, were tested for B12 and folate deficiencies. Both were confirmed as having B12 deficiency. None of them had folate deficiency.

### Microcytic anaemia with high red cell count

Blood films were reported in 24 out of the 91 with microcytic anaemia and high RCC. As hypothesised, all 24 had features of minor hemoglobinopathy. Independently of them, 19 underwent HPLC as a part of this study and 17 had undergone HPLC previously. Beta Thalassemia trait was diagnosed in 35 of them while the other had Haemoglobin SE disease (HbSE). Thus, with a 100% positive predictive value observed (with 95% CI, 100–100%), we estimated that all 91 participants with microcytic anaemia and high RCC had minor hemoglobinopathies.

### Anaemia with normocytic hypochromic cells

Seven randomly selected participants (out of the 84) underwent peripheral blood film analysis. Four were having changes suggestive of early iron deficiency. Two had normal blood film reporting, and one had megaloblastic changes. Independent to this, five had records of past HPLC testing, and all five were negative for minor hemoglobinopathies.

### Macrocytic Anaemia

Only one out of the two cases of macrocytic anaemia had a peripheral blood film reporting. The blood film showed early megaloblastic changes. However, her serum B12 level was 321 pg/ml, and her serum folate level was 29.2 ng/ml.

### Non-anaemic pregnant women

Peripheral blood film was analysed in a random sample of 143 non-anaemic women. Minor hemoglobinopathy, early iron deficiency and iron deficiency was suggested in 7 (4.9%), 12 (8.4%) and 2 (1.4%) of the blood films, respectively, and the others (122, 85.3%) did not show an abnormality.

Of the 2676 non-anaemic women, 145 had HPLC reports (136 had records, and nine underwent testing during the current study), and 12 of them were confirmed to have minor hemoglobinopathies.

### Subsample analysis

213 random first trimester pregnant women from the total sample were selected for sub-sample analysis for serum ferritin. Anaemia prevalence in this sub-sample was 14.5% (n-31).

### Serum ferritin

The distribution of serum ferritin was skewed to the right (skewedness 2.78). The mean and median serum ferritin level among 213 first trimester women was 67.6 (SD 55.5) and 56.8. Iron deficiency (serum ferritin < 30 ng/ml) was seen in 20.2% (n-43, CI 15.4–26.1). Serum ferritin was less than 15 ng/ml in nine (4.2%) participants. Respectively, 164 (77%) and 6 (2.8%) of participants had s. ferritin between 30 to 199.9 ng/ml and ≥ 200 ng/ml. Of those with iron deficiency, 13 (30.2%) had anaemia, while 30 (69.8%) were non-anaemic. Conversely, 41.9% of anaemic and 15.7% of and non-anaemic participants were iron deficient.

Serum ferritin level and red cell indices-based evidence on possible iron deficiency shows poor agreement (Table 2). Ferritin was low in only a quarter of women with either microcytic or hypochromic red cell indices.

**Table 2:** Compatibility of serum ferritin and red cell indices-based evidence of iron deficiency in early pregnancy

Serum Ferritin	Anaemic		Non anaemic	
	MCV^a^/ MCH ^b^ low	MCV/MCH normal	MCV/ MCH low	MCV/MCH normal
	N (%)	N (%)	N (%)	N (%)
Low	10 (45.5)	3 (33.3)	17 (21.3)	11 (11.3)
Normal	12 (54.5)	6 (66.7)	63 (78.8)	86 (88.7)

^a^Mean Corpuscular Volume, ^b^Mean Corpuscular haemoglobin

### Serum B12 and folate

Of these 31 anaemic pregnant women, 21 came for serum vitamin B12 and folate testing. Both parameters had wide variation among the participants.

Serum Folate levels ranged between 5 and 14 ng/ml. Median, Mean and SD were 14 g/ml, 19 ng/ml and 10, respectively. Folate deficiency (< 4 ng/ml) was not observed among participants. However, one (4.8%) had a folate level below 5.9 ng/ml (risk of deficiency), and eight (38%) had high folate levels above 20 ng/ml.

Serum Vitamin B12 level of the sample varied between 154 and 721 (pg/ml). The Median was 301 pg/ml, mean was 359 pg/ml (SD178). B12 deficiency (B12 < 203 pg/ml) was observed among 5 (23.8% CI 10.6–45.1). Another five had B12 levels between 203 to 300 pg/ml, while 11 (52.4%) had B12 levels more than 300 pg/ml.

**Fig. 3:**
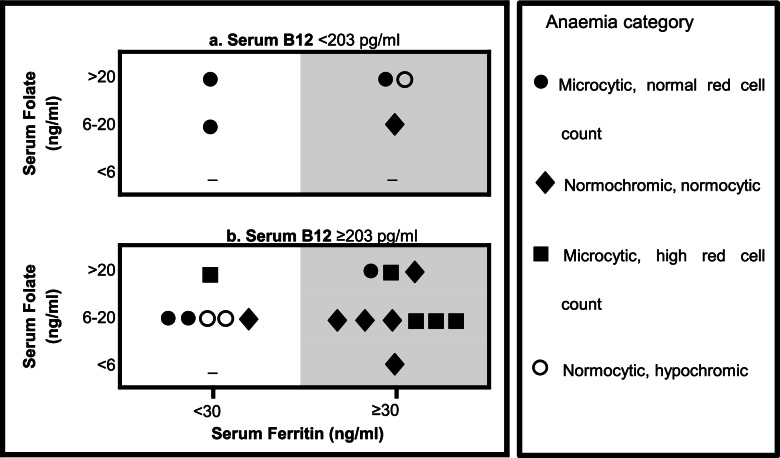
Red cell indices, Serum Folate level, and iron and B12 deficiency status in the subsample of 21 first trimester anaemic pregnant women. (Panel a – among vitamin B12 deficient participants, panel b among participants with normal Vitamin B12 levels)

None of those with B12 deficiency had Macrocytic anaemia (according to red cell indices). However, the peripheral blood film of one of them showed oval Macrocytes. Co-existing iron deficiency was seen in two. Three of the five had high folate levels. The distribution of B12, folate and ferritin levels and types of anaemia are shown in Fig. 3.

### Other systemic diseases

Small percentages of anaemic pregnant women had previously diagnosed thyroid (*n* = 9, 2.1%), liver (*n* = 3, 0.7%), renal (*n* = 2, 0.4%), or autoimmune diseases (*n* = 2, 0.4%). Fissure’s exact test showed that prevalence of anaemia was not significantly different between groups with and without a diagnosed thyroid (p – 0.87), liver (p − 0.06), renal (p-1) or autoimmune disease (p-0.21).

In summary, we estimated that at least 23.3% (*n* = 105) of first trimester anaemic women have minor hemoglobinopathies. The prevalence of iron deficiency, B12 deficiency and Southeast Asian ovalocytosis were estimated as 41.9% (95% CI 26.4–59.2), 23.8% (CI 10.6–45.1), and 0.9 95% CI 0.3 to 2.3%) of first trimester anaemic women respectively. Folate deficiency was not observed among them.

## Discussion

Improving maternal health beyond numbers requires an in-depth understanding of the common conditions and incorporating those into the health systems. Public health guidelines on anaemia, a well-known underlying cause of maternal morbidity and mortality, has not been updated for many years due to a lack of understanding of underlying factors. This large population-based study examines the aetiologies of anaemia in pregnancy with a macro lens, revealing previously unidentified aspects to inform public health policy.

Using a population-based, representative sample [31, 46], including 86% of the reference population [31, 46], we demonstrated that the anaemia prevalence among first trimester pregnant women in the district was 14.4% (95% CI 13.2–15.7). This prevalence can be classified as a problem of mild public health significance [47]. Even though the reported national prevalence of anaemia in pregnancy is high (29.1%) [31], first trimester anaemia prevalence reported in studies during the last decade are consistently low (16.9 to 14.4%) [48, 49]. The absence of severe anaemia is also compatible with findings from previous studies in Sri Lanka [48, 50] showing the success of the country’s public health interventions to control anaemia across the lifecycle. Of note, a significant proportion (12.7%) had haemoglobin > 13 g/dl, a condition which has shown to be associated with adverse pregnancy outcomes [51, 52] and this needs to be followed up further.

### Minor Hemoglobinopathies

Microcytic anaemia is generally associated with iron deficiency and thalassemia trait. Different red cell indices-based markers are available for minor haemoglobinopathy screening in people with microcytic anaemia. According to current evidence, the RBC index is one of the best amongst them [40]. The sensitivity and specificity of these markers vary in different populations. With the available HPLC confirmed results, we have shown that the positive predictive value of the RBC index in the study population is 100%, an observation supported by the blood film analysis as well. Combining this with already tested and confirmed cases that do not have a high RBC index, a significantly high prevalence of minor hemoglobinopathies (at least 23%) was identified among anaemic pregnant women in this population.

We did not test for alfa thalassemia in the present study, and the majority of the confirmed cases were beta-thalassemia trait. However, the global prevalence of alfa thalassemia is higher than that of beta [53]. Alfa thalassemia carrier status is estimated to be present in 20.7% of the world population [28]. A study conducted among anaemic first trimester women from a different location in Sri Lanka has also shown that the prevalence of beta-thalassemia trait was 15%, while the prevalence of alfa thalassemia trait was 17% [54]. HbE and beta-thalassemia trait were reported in 1 and 2% of school children in Anuradhapura [53]. Published data on other haemoglobin variants and the findings from the current study suggest that the contribution of minor hemoglobinopathies as an etiological factor for anaemia in pregnancy in this population is extremely higher than expected. Findings of the study (before publication) were extensively discussed among national-level policymakers. As a result, the national maternal care programme introduced a new policy to screen all anaemic pregnant women with microcytic hypochromic anaemia for minor haemoglobinopathies [55].

### Iron deficiency

Serum ferritin is effective to identify iron deficiency [37]. Serum ferritin can be falsely elevated due to inflammation and should ideally be combined with an inflammatory marker such as C Reactive Protein (CRP) [37]. However, since variable ferritin cut offs are being employed to determine iron deficiency, we selected a higher cut off value of 30 ng/ml, which is the WHO recommendation for pregnant women with evidence of inflammation [37, 38]. This cut off will increase the sensitivity to detect iron deficiency despite inflammation.

Iron deficiency is well-documented among Sri Lankan pregnant women. Early studies have shown very low mean ferritin levels (12.39 ng/dl) and very high iron deficiency prevalence (69% with 12 ng/dl cut off) during pregnancy [56]. Probably due to public health interventions and improved socioeconomic status, iron deficiency has shown a decline. For example, recent hospital-based studies show that iron deficiency prevalence among pregnant women was 21.2%(Colombo, 2009) [57] and 36.9% (Mahamodara, 2015) [58]. In an island-wide sample (2015), iron deficiency varied between 10% in Anuradhapura and 42.5% in Jaffna [48]. Prevalence of iron deficiency anaemia among first trimester women was 6.4% in the hospital-based study in Colombo (13 out of 203 had ferritin < 20 ng/dl), 15.9 in a community clinic-based study in Colombo (10 out of 90 had ferritin < 30 ng/dl) and 2.4% in the national-wide sample (3 out of 127 had ferritin < 15 ng/dl) [49]. The current study presents the highest number of ferritin estimations in first trimester pregnant women of Sri Lanka, the prevalence of iron deficiency was 20.1%, and iron deficiency anaemia was 6.1%. Though Anuradhapura has a higher anaemia prevalence than most other districts, this cannot be attributed to a higher iron deficiency prevalence. Other studies from LMIC also have shown that most anaemic pregnant women are not iron deficient [59]. However, 41.9% of anaemic women have iron deficiency. Hence prevention and treatment of iron deficiency remain a priority for controlling anaemia in this community.

Even though the ferritin levels suggest a comparatively low prevalence of iron deficiency, the majority of the participants have microcytic anaemia (35.9% when high RCC is excluded) and normocytic hypochromic anaemia (18.4%). These indicators are usually attributed to iron deficiency [60]. Previous studies in this population also have shown that the Serum ferritin based iron deficiency prevalence is lower than suggested by other evidence such as red cell indices and peripheral blood film analysis [61]. This observation should be investigated further.

### Vitamin B 12 and folate deficiency

Two previous studies conducted in more urban settings in Sri Lanka; among school dropped out girls (15 to 19 years old) and nonpregnant, nonlactating females (15 to 30 years old), have shown a low prevalence of B12 deficiency (1.6 and 0.44% respectively) [62, 63]. To our knowledge, this is the first study on B12 deficiency among pregnant women in Sri Lanka. Our data show that B12 deficiency is an important contributor to anaemia during early pregnancy in this population as 23.8% of anaemic first trimester women were reported to have B12 deficiency, which amounted to 2.2% of prevalence among all pregnant women. Only about 28% of B12 deficiency is manifested with haemoglobin changes [64]. We only assessed the B12 status of anaemic women. Vitamin B12 deficiency may exist without anaemia, in which case the prevalence of deficiency among pregnant women will be higher than the reported value. Poor diet could be linked to the significant prevalence of deficiency observed since vitamin B12 consumption is below the Estimated Average Requirement in 42.1% of Sri Lankans [65]. Pregnancy may lead to a 25 to 30% reduction in serum B12 levels [66]. But this change is minimum in the first trimester as the B12 level is gradually reduced to reach its lowest at term [67, 68]. Therefore, the observed B12 levels likely indicate an actual deficiency state rather than an arbitrary change due to pregnancy.

B12 deficiency during pregnancy is associated with low birth weight, preterm delivery and neural tube defects in addition to maternal anaemia [69, 70]. There is a probable Association with preeclampsia as well [70]. Further assessment of the B12 status in pregnant women in the country should be undertaken urgently and universal supplementation during pregnancy should be considered.

Folic acid deficiency has been reported among reproductive-age females of the country [62, 63]. However, we did not find anyone with folic acid deficiency, possibly because most participants had consumed Folic acid supplements during the pre-conceptional and antenatal period*.* It is noteworthy that a significant proportion, including 60% of the B12 deficient participants, had high folate levels. Since evidence suggests that high folic acid levels may exaggerate the effects of B12 deficiency, it is worthwhile to reconsider the strategy of supplementing high doses of folic acid (e.g., during preconception and first trimester) without B12 supplementation [27, 71].

Folate or B12 deficiency is expected to cause macrocytic anaemia [43]. Studies, including the current, show that macrocytic anaemia is uncommon among first trimester pregnant women from this region [50]. We observed megaloblastic changes (in the peripheral blood films) or low serum B12 levels in several participants with microcytic or normocytic anaemia. This observation may be attributed to the presence of mixed deficiency [72]. Therefore, using the presence of macrocytic anaemia to screen for pregnant women needing further assessment and treatment in the line of B12 or Folate deficiency is not advisable.

### South-east Asian Ovalocytosis

South-East Asian Ovalocytosis has been reported from Sri Lanka previously. This is the first study reporting the significance of south-east Asian ovalocytosis in Sri Lankan pregnant women [73, 74]. One per cent of anaemic first trimester women had this condition. Cases were identified by selective screening of pregnant women with normocytic normochromic anaemia. We can attribute the anaemia in these women to the presence of membrane disorder as random testing did not find any cases among non-anaemic women.

### Systemic diseases and other conditions

We did not find evidence suggesting thyroid, liver, renal or autoimmune diseases significantly contributing to maternal anaemia in this community, perhaps due to the low number of reported cases. Even though Malaria and HIV contribute to significant numbers of anaemia in pregnant women around the globe, we did not test for them in the current study as the prevalence of both conditions in Sri Lanka are negligible [75, 76].

This study has several limitations. Some aetiologies were assessed based on an evidence-based algorithm instead of a universal screening due to funding restrictions. Further, the prevalence of alfa thalassemia was not covered in this study. We established that B12 deficiency is prevalent in this community, but the sample tested was small, and studies with a larger sample are required to obtain proper estimates. Such assessments should include other micronutrient deficiencies such as vitamin B6 and Zinc as well.

The findings of this study from rural Sri Lanka calls for a re-evaluation of current universal strategies implemented in preventing anaemia in pregnancy. Even in low- and middle-income settings where iron deficiency is prominent, and the first line of management is doubling the iron dose, exploring the underlying causes of maternal anaemia will help improve mothers and babies health.

## Conclusion

We have shown that iron deficiency remains a major cause of anaemia in pregnant women despite already implemented preventive strategies across the lifecycle. Bio-socio-cultural reasons for the continued predominance of iron deficiency in this community need to be evaluated further. Minor hemoglobinopathies significantly contribute to anaemia in pregnancy. The role of minor hemoglobinopathies in maternal anaemia in other LMIC’s needs to be further investigated. Application of sound evidence on aetiologies of anaemia in pregnancy will be beneficial in reviewing current policies and strategies in LMICs for further improvement of health in mothers and babies.

## Data Availability

The datasets used and/or analysed during the current study are available from the corresponding author on reasonable request.
